# *StableClim*, continuous projections of climate stability from 21000 BP to 2100 CE at multiple spatial scales

**DOI:** 10.1038/s41597-020-00663-3

**Published:** 2020-10-12

**Authors:** Stuart C. Brown, Tom M. L. Wigley, Bette L. Otto-Bliesner, Damien A. Fordham

**Affiliations:** 1 The Environment Institute and School of Biological Sciences, University of Adelaide, South Australia, 5005 Australia; 2 Climate and Global Dynamics Laboratory, National Center for Atmospheric Research, Boulder, CO 80307-3000 USA

**Keywords:** Biogeography, Ecological modelling, Macroecology, Palaeoclimate, Projection and prediction

## Abstract

Paleoclimatic data are used in eco-evolutionary models to improve knowledge of biogeographical processes that drive patterns of biodiversity through time, opening windows into past climate–biodiversity dynamics. Applying these models to harmonised simulations of past and future climatic change can strengthen forecasts of biodiversity change. *StableClim* provides continuous estimates of climate stability from 21,000 years ago to 2100 C.E. for ocean and terrestrial realms at spatial scales that include biogeographic regions and climate zones. Climate stability is quantified using annual trends and variabilities in air temperature and precipitation, and associated signal-to-noise ratios. Thresholds of natural variability in trends in regional- and global-mean temperature allow periods in Earth’s history when climatic conditions were warming and cooling rapidly (or slowly) to be identified and climate stability to be estimated locally (grid-cell) during these periods of accelerated change. Model simulations are validated against independent paleoclimate and observational data. Projections of climatic stability, accessed through *StableClim*, will improve understanding of the roles of climate in shaping past, present-day and future patterns of biodiversity.

## Abstract (summary)

**Table:** 

Measurement(s)	climate change • climate • temperature of air • volume of hydrological precipitation
Technology Type(s)	computational modeling technique • digital curation
Factor Type(s)	timing of temperature and precipitation estimates
Sample Characteristic - Environment	climate system
Sample Characteristic - Location	Earth (planet)

Machine-accessible metadata file describing the reported data: 10.6084/m9.figshare.12831935

## Background & Summary

A stronger understanding of the relationships between past climatic change and contemporary geographic distributions, and abundances of species, and ecosystem structure and function, can improve capacities to anticipate, and potentially manage responses of biodiversity to rapid future climate change, and global change more generally^1,2^. Interdisciplinary approaches that combine macroecological models with inferences from paleoclimate simulations, paleoecology, and paleogenomics are opening windows into climate–biodiversity dynamics during the late Quaternary^3,4^. This research has shown that a primary factor constraining the distributions and diversity of species at macro-scales is climate stability^5–7^, with hotspots of biodiversity often occurring in regions that have experienced stable temperatures and variable rates of precipitation during the late Pleistocene and Holocene^8–11^.

Unravelling the mechanisms that have shaped ancient and current-day patterns of biodiversity requires spatially detailed and temporally consistent datasets of paleo climatic change^4^. While there is a growing library of high spatial and temporal resolution paleo climate datasets available to researchers^12–14^, issues relating to spatiotemporal coverage and continuity persist. Furthermore, a lack of paleo climate simulations harmonised (i.e., consistently spatially and temporally blended to) with independently derived future projections is preventing a wider integration of paleo-archives and paleo perspectives in model projections of future biodiversity change. Missed opportunities include providing the context and tools needed to guide conservation decisions regarding desired states of ecological systems under global warming^15,16^. Although there have been attempts to overcome this problem^17–19^, a lack of spatial and temporal continuity in simulations that extend from the past into the future remains^14^.

Blended data on centennial trends and variability of temperature and precipitation are needed to calculate consistent spatiotemporal changes in climatic stability from the Last Glacial Maximum to the end of the 21^st^ century, enabling the eco-evolutionary impacts of climate change to be quantified^8^. Here we provide continuous gridded global-scale estimates of centennial trend, variability, and signal-to-noise ratio (SNR) in temperature and precipitation between 21,000 B.P. and 2100 C.E. We do this by harmonising, at 2.5° spatial resolution (~278 km at the equator), three distinct data sets: paleoclimate simulations from the TraCE-21ka coupled atmosphere-ocean-general-circulation-model (AOGCM)^20,21^, historical runs from 19 CMIP5 AOGCMs, and future projections from the same CMIP5 19 AOGCMs under 4 Representative Concentration Pathways (RCPs)^22,23^.

We use pre-industrial control runs from CMIP5 AOGCMs to define thresholds that can be used to identify centuries of past and future rapid high magnitude temperature change at global and regional scales. We do this separately for terrestrial and ocean realms, for distinct IPCC AR5 climatic regions^24^, and for terrestrial zoogeographic realms^25^. These thresholds enable users to subset *StableClim* to periods of rapid warming (or cooling) at global and/or regional scales^25^, allowing rapid climate change events^26^ that occurred in the past to be identified in space and time and compared directly with those projected for the future. Regions that experienced past climate shifts that are of similar magnitude to future forecasts provide locations where geohistorical data can be used to better derive and strengthen conservation management and policy through improved knowledge of biotic responses to climatic stressors^2^, and for connecting theory to the on-ground design and implementation of effective measures to protect biodiversity^27^.

*StableClim* also includes continuous coverage of gridded monthly-mean temperature and total monthly precipitation between 1850 and 2100 at monthly time-step with 2.5° × 2.5° spatial resolution. When combined with PaleoView^28^ this provides users with more than 21,100 years of harmonised monthly temperature and precipitation climatic data. This feature, allows end-users to generate alternative measures of climate stability, including climate velocity^29^, for the past and the future at temporal scales different to those provided in *StableClim*.

## Methods

### Overview

**Fig. 1:**
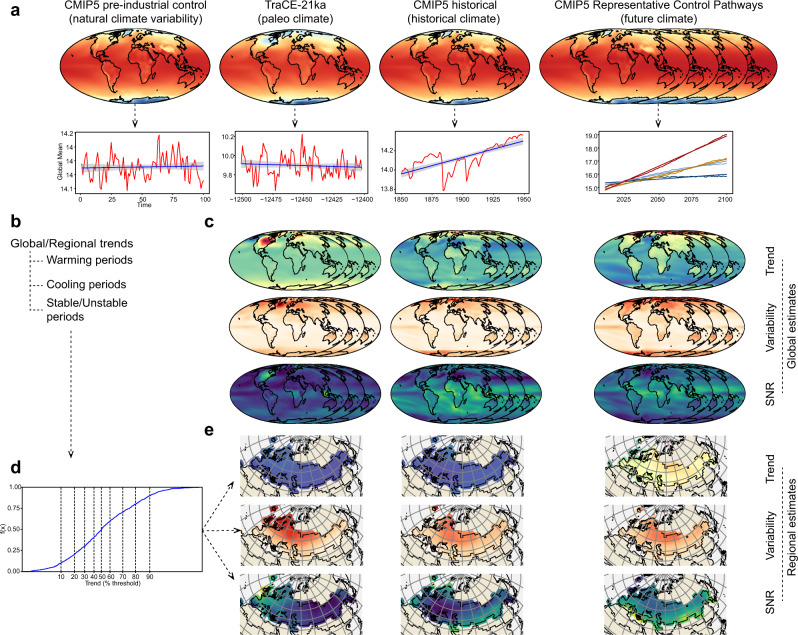
Overview of the *StableClim* database. Simulated climate data for temperature and precipitation for pre-industrial, past, historical, and future climates come from 19 CMIP5 climate models (**a**). Paleo climatic conditions come from the TRaCE-21ka simulation. One-hundred-year trends in mean temperature for the past, historical, and future climates are provided at global and regional scales (**b**). Gridded datasets (*n* = 10,368 cells) of trend, variability, and signal to noise-ratio for the past, historical, and future climates are provided at global scales (**c**). Thresholds are used to identify past and future periods of rapid warming and cooling and stable climatic periods based on natural variability from the pre-industrial control runs (**d**). Thresholds are applied to the continuous grid-based trends, variability, and signal-to-noise ratio (21,000 B.P. to 100 C.E.), allowing estimates of climatic stability during specific periods in Earth’s history and potential future (**e**).

An overview of the design of *StableClim* is provided in Fig. 1. Broadly, 19 Atmosphere-Ocean General Circulation Models (AOGCMs), from the Coupled Model Inter-comparison Project phase 5 (CMIP5)^30^ were used to calculate continuous estimates of trend, variability, and signal-to-noise ratios (SNR) in pre-industrial control, historical, and future climates under four different emissions scenarios. Simulated climate data from the TraCE-21ka^20^ experiment was used to calculate the same metrics for paleo climates since the Last Glacial Maximum. Global and regional estimates of trend for pre-industrial control temperatures can be used to identify past and future extreme centennial conditions.

### Pre-industrial, paleo, historical, and future climate data

#### Data access

Pre-industrial, historical, and future climate datasets with global coverages of modelled monthly-mean surface temperature, and monthly precipitation were extracted from the CMIP5 Earth System Grid Federation data portal (https://esgf-node.llnl.gov/projects/esgf-llnl/) using customised bash scripts (available from https://github.com/GlobalEcologyLab/ESGF_ClimateDownloads). Paleoclimate data from the TraCE-21ka experiment was extracted from PaleoView^28^ at a monthly time-step for the period 21,000 B.P. to 100 B.P. (1850 C.E.).

We used four different modelled climate datasets to generate the climate data compiled in *StableClim* (Fig. 1):

1. Pre-industrial control runs for 19 AOGCMs from CMIP5 were used to quantify natural climate variability,
2. Paleoclimate simulations from the TraCE-21ka experiment were used. TraCE-21ka simulations were done with the Community Climate System Model ver. 3 (CCSM3)^31,32^,
3. Historical simulations (1850–2005 generally) from the same 19 CMIP5 AOGCM’s that were used to generate the pre-industrial control climates^25^,
4. Representative Concentration Pathway (RCP)^22,23^ 2.6, 4.5, 6.0, and 8.5 runs for the same 19 CMIP5 AOGCM’s used to generate pre-industrial control climates.

While the chosen RCP scenarios are four of hundreds of future climate scenarios currently available, they span a wide range of possibilities. RCP 8.5 and RCP 6.0 are commonly thought to represent “Business As Usual” scenarios (i.e., with no new mitigation policies), whilst RCP 4.5 and RCP 2.6 are within the spectrum of mitigation policy scenarios.

#### Pre-industrial climate

Pre-industrial control runs are multi-century unforced climate simulations, where the initial model conditions are set based on atmospheric gas concentrations prior to large-scale industrialisation^25^. They have non-evolving boundary conditions (e.g. non-evolving land use and greenhouse gas concentrations) relevant to the chosen start year^25^ and ignore natural forcing effects such as those caused by variations in the Sun’s output, and relatively short-term cooling of explosive volcanic eruptions. They therefore capture only internally-generated variability. We elected to use only the first realisation (r1i1p1) from each model for the pre-industrial control runs as all models, with the exception of the Community Climate System Model ver. 4 (CCSM4)^33^, only had a single realisation (i.e. a single set of initial conditions). The additional pre-industrial realisations (r2i1p1 and r3i1p1) for the CCSM4 model were too short to be used. The shortest duration pre-industrial control run used in this analysis was 240 years (HadGEM2-CC; see Online-only Table 1).

#### Paleoclimate

The TraCE-21ka experiment was chosen to represent paleo-climate conditions because (i) the data are available at a high temporal (monthly) and moderate spatial (2.5° × 2.5°) resolution with global coverage^28^; and (ii) the model has been independently validated at multiple temporal and spatial scales^28,34–36^. These independent validations have shown that the TraCE-21ka model effectively reconstructs important regional-to-global paleoclimatic fluctuations during the last deglaciation event^28,34–36^ and accurately simulates present-day climate patterns^28^.

#### Historical climate

The historical simulations cover the period 1850–2005 (in some extended cases they continue to 2012), with the beginning of the modelling period occurring before significant anthropogenic forcing and climate change. The historical climate simulations allow simulated climatic conditions to be validated against observed datasets^25^. The historical simulations differ from the pre-industrial control conditions as they are forced by observed atmospheric composition changes and aerosol emissions (for both anthropogenic and natural sources) and include the effects of solar irradiance variations and major volcanic eruptions, and time-evolving land and sea-ice cover. All available model realisations were used for the historical period as there can be significant differences in trends due to internal climate variability in the models^37^. We chose to include all model realisations, as there is no way to determine which of the realisations should be preferred over others, and each realisation will lead to a slightly different climate state^38^. For example, all members within an ensemble of historical runs (e.g. CCSM4 r1i1p1, r2i1p1, r3i1p1) are forced in the same way, but each is initiated at a different point in the pre-industrial control run^25^. The differences in initial conditions result in different trajectories, and multi-realisation averaging reduces this “noise”^39^.

#### Future climate

The RCP scenarios describe a set of possible climate outcomes as a result of changes in emissions, land use, and sea-ice developed specifically to allow assessment of future climates over a wide range of warming scenarios^23^. The RCP numerical designation indicates the radiative forcing level reached at the end of the century (e.g. RCP 8.5 is a high emissions warming scenario with radiative forcing level reaching approximately 8.5 W/m^2^ by 2100)^23^. The two intermediate scenarios feature a peak-and-stabilise scenario, whereby the radiative warming peaks at the given level before stabilising by 2100 (RCP 4.5) or shortly thereafter (RCP 6.0). The low emissions RCP 2.6 scenario has radiative forcing peaking in the middle of the 21^st^ century before decreasing to an eventual nominal level of 2.6 W/m^2^ ^23^. As with the historical climate simulations, assessment of the RCP scenarios utilised all available model realisations to reduce inter-model noise in the ensemble average.

#### Pre-processing of climate data

To address the different temporal extents and spatial resolutions of the AOGCMs used to generate *StableClim*, a number of pre-processing steps were required to ensure that the different datasets were consistently blended to have an adjoining timeframe for the period of interest, and that the data were on a spatially consistent grid. Pre-processing was performed using the Climate Data Operators (version 1.9.3) software^40^.

Modelled years for TraCE-21ka simulations were constrained to the period 21,000 B.P. to 100 B.P. (1850 C.E.) to limit the influence of wide scale industrialisation on the paleoclimate simulations^41^. The start-date for the CMIP5 historical simulations is 1850. An end-date of 2005 was chosen because of the low number of models (*n* = 3; BCC-CSM1.1, CNRM-CM5, and MIROC5) with simulations extending beyond this time period. The RCP scenarios simulate possible future climates between 2005 and 2100 and are initialised using the climate conditions at the end of the historical period (2005). As the RCP simulations are essentially continuations of the historical simulations^23^ and we needed to have continuous centennial trends between the paleo, historical, and future periods, we temporally harmonised the historical and RCP simulations. Following Santer, *et al*.^42^ we spliced the historical simulations to the beginning of the RCP simulations ensuring that the realisations matched so that there were no differences in simulation forcings (e.g. CCSM-4 historical r1i1p1 was matched to CCSM-4 RCP r1i1p1). To account for intra-model variability, each model (e.g. CCSM-4) was then averaged across all realisations within that model to produce a multi-realisation model average.

To enable spatially consistent comparisons with the TraCE-21ka simulation, the CMIP5 data were re-gridded to a 2.5° × 2.5° (latitude/longitude) global grid using bilinear interpolation. Re-gridding of the CMIP5 datasets to match the resolution of the TraCE-21 data using bilinear interpolation was chosen because (i) the source and destination grids were rectilinear, (ii) precipitation and temperature in the climate models varies smoothly spatially, and (iii) bilinear interpolation (more or less) retains the integrity and limitations of the original model output data, where orography is highly smoothed relative to the real-world^28^. Furthermore, the 2.5° × 2.5° grid cell resolution corresponds to the resolution of the TraCE-21ka data as documented in *PaleoView*^28^, (bilinearly downscaled to 2.5° × 2.5° from its nominal original resolution of ~3.75°^28^), and the resolution of projections from MAGICC/SCENGEN^43^. Surface temperatures and precipitation were then converted to °C (from Kelvin) and mm/year (from kg m^2^ s^1^) respectively.

#### Calculating trends in global mean temperature

**Fig. 2:**
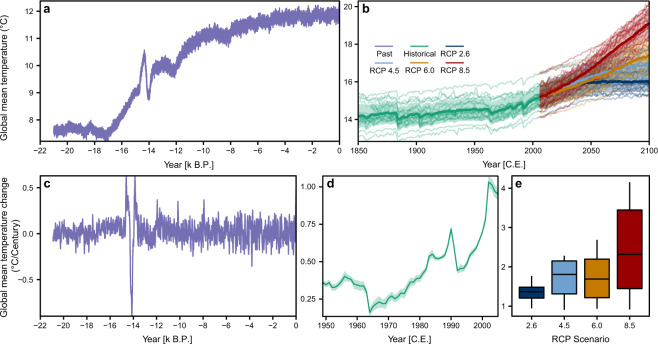
Annual global mean temperature and trend in global mean temperature from the Last Glacial Maximum to the end of the 21^st^ century. The Global mean temperature during the past as simulated by TraCE-21ka (**a**), and spliced historical/future climate simulations to 2100 (**b**). Trends in global mean temperature for past (**c**), historical to 2005 (**d**), and for the future under four different RCP scenarios (**e**). The individual lines in **b** show the multi-realisation model averages, with the bolder lines showing the multi-model ensemble average for the respective scenario. The shaded areas in **b** and **d** show the multi-model variability in global mean temperatures and trend estimates (±1 S.D.). The timesteps in **c** and **d**, show the end-year of the century window (e.g. 1950 = window from 1851:1950 C.E.). Values in **e** show slopes for 2006 to 2100 C.E. Note that the y-axis differs between all plots.

Continuous estimates of trends in global-mean temperature through time allow comparisons of rates of change during key periods in Earth’s history and those projected for the future (Fig. 2). Pre-industrial control-runs can be used as a baseline for identifying high magnitude and rapid changes in global mean temperature (“extreme” events) that occurred in the past and likely to occur in the future^8,44^ Accordingly, we determined linear trends in area-weighted global-mean surface temperature associated with natural variability^45^ for maximally overlapping century long windows for each of the CMIP5 pre-industrial control runs. This means that for a time series 1,2,3 … N, the 100-year windows would be years 1–100, 2–101, 3–103, etc. We calculated weighted global mean temperature for each year using the cosine of the latitude of the grid-cell centroids as weights.

Trends for annual area-weighted global-mean temperatures were then calculated using Generalised Least Squares (GLS) regression with AR(1) errors. The GLS models were calculated using the ‘*nlme*’ package^46^ for R (version 3.5.1)^47^. GLS regression with an AR(1) error structure was chosen to minimise any effect of temporal auto-correlation in the model residuals^46^. The resulting global natural trends (i.e., the slope of the regression) for surface temperature were used to generate a multi-model, pre-industrial cumulative distribution function (CDF) using signed slopes.

Because the number of years varied between pre-industrial control runs from different AOGCMs (Online-only Table 1) we used a bootstrap procedure to ensure that all models had equal weights in the CDF (i.e. we did not want to bias the CDF towards models that had longer simulations, or higher/lower modelled global-mean temperatures). The bootstrap procedure for each model involved first selecting the slopes for all overlapping windows for a given model, and then randomly selecting slopes from that model (with replacement) equal to the difference between the number of overlapping windows for the model, and the maximum number of overlapping windows across all models (n = 952). For example, for model ACCESS 1.3 which has 500 years of simulated pre-industrial control conditions, the maximum number of overlapping centennial windows is 401. For the bootstrap procedure, slopes for the 401 overlapping windows were first selected, before 551 slopes were then selected randomly with replacement, giving 952 slope values. The bootstrap procedure was repeated 1000 times for each of the 19 models before building the CDF. This process ensured that all intra-model variability was accounted for, while the effect of longer simulation runs was eliminated.

For the past (21k B.P. – 1850 C.E.) and spliced historical/future climate (1850–2100 C.E.) we calculated trends in global-mean temperature using the methods described above (Fig. 2). However, we did not use a bootstrap approach because for the paleo period we only had a single simulation (TraCE-21ka), and the historical and future simulations were a multi-model ensemble average, subset to a consistent temporal window which negated the need for a bootstrap. Multi-model averages for the spliced historical/future climate were calculated by averaging across all multi-realisation model averages (n = 19, see *Pre-processing of climate data*). Whilst this approach may bias the results of models that have multiple realisations (as the intra-model variability is effectively reduced by averaging across realisations), it has been shown that the performance of multi-model ensemble averages improves with an increase in models, not realisations^48^. This process allowed us to effectively calculate robust measures of global mean temperature that accounted for intra- and inter-model variability^37,42,49^.

#### Calculating trends in regional mean temperature

We quantified linear trends in area-weighted regional-mean surface temperature associated with natural variability for maximally overlapping century long windows for each of the CMIP5 pre-industrial control runs. As for trends in global mean temperature (see above), we also calculated trends in regional-mean temperature for the past (21k B.P.–1850 C.E.) and spliced historical/future climate (1850–2100 C.E.). The regions were defined by 18 distinct IPCC AR5 climatic regions^24^, 19 Wallace Zoogeographic zones^25^, and 11 zoogeographic realms^25^. Temperatures were extracted for grid-cells inside the boundary of the region. Weights for the regions were calculated as above. The 18 IPCC AR5 climatic regions are an amalgamation of the terrestrial regions defined by Working Group 1 for the IPCC Fifth Assessment Report^50^. The Wallace Zoogeographic zones and realms follow Holt, *et al*.^25^ although the Polynesian zone was removed due to its small size (average island size in the Polynesian zone is ~118 km2, or approximately 0.15% of the area of our grid-cells). A geopackage of the IPCC regions, the Wallace zones, and the zoogeographic realms we used is available in *StableClim*.

#### Identifying thresholds of extreme climate change

To identify periods of rapid, medium and slow climate change at global and regional scales, we used the ensemble averaged bootstrapped pre-industrial CDF of trends in global/regional mean temperature at 1, 2.5, and 5% increments (e.g. 1%, 2.5%, 5%, 10%, 15%…90, 95%, 97.5%, 99%) to identify rates of change that correspond to different “thresholds” of stable climate (at lower thresholds) or rapid climate change (at higher thresholds)^8,44^. Based on Fordham, *et al*.^44^, we define a stable climate as having low rates of centennial change (i.e. low trend values), and an unstable climate as having high rates of centennial change. Notably, these definitions do not however preclude high inter-annual variability (i.e. high frequency climate instability) in ‘stable’ conditions or low inter-annual variability for ‘unstable’ conditions. The 90^th^ percentile of the pre-industrial CDF has previously been used to identify periods of change in global mean temperature that had high absolute (i.e. unsigned) rates of climate change since 21,000 B.P.^8,44,51^.

Thresholds were calculated at a range of scales and for different regions and realms. For climate focused studies, thresholds are provided at regional scales using the IPCC AR5 climatic regions described above. For biogeographical or ecological focussed work, thresholds are provided at two scales: (i) 19 smaller scale Wallace Zoogeographic zones and (ii) 11 broader scale terrestrial zoogeographic realms, both described above.

#### Calculating local trends, variability, and SNR

**Fig. 3:**
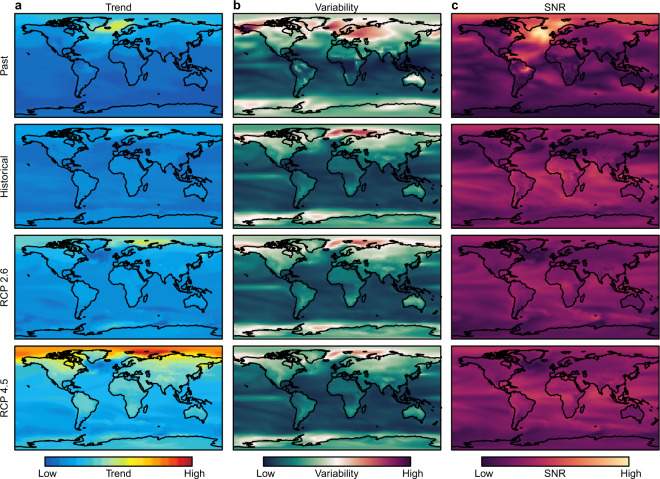
Maps of trend, variability, and signal to noise ratio (SNR) for temperature during periods of extreme global warming in the ocean and on land (≥90^th^ percentile from pre-industrial conditions). Maps of centennial trend (**a**), inter-annual variability (**b**), and SNR (**c**). Rows represent rapid global warming events at different time periods/climate scenarios. Past = Bølling–Allerød (14.7-14.2k B.P.^69^); Historical = 1850 C.E.–2005 C.E.; RCP 2.6 & 4.5 = Representative Concentration Pathways 2.6 and 4.5 for 2001 C.E.–2100 C.E. Maps of the past and historical conditions are mean estimates for overlapping century windows during the relevant periods.

Trend and the variability around the trend are the primary components of climate stability^44^, and they provide a distinction between low frequency (long term trend) and high frequency (inter-annual) climate stability. For both temperature and precipitation, we calculated ‘local’ measures of centennial linear trend (i.e. inter-centennial variability; low frequency climate in/stability) for each grid-cell (*n = *10,368 cells) for the paleo, and the spliced historical/RCP simulations (Fig. 3). We also calculated grid-cell estimates of variability (i.e. inter-annual variability; high frequency climate in/stability), where variability was defined as the standard deviation of the residuals about the local trend^52^.

We also calculated a signal-to-noise ratio (SNR = abs(trend)/variability)^53^, for both temperature and precipitation. We opted to consider SNR in addition to trend and variability, because the SNR is a composite measure of the trend given background variability (Fig. 3). Furthermore, the SNR can be useful in comparing climate stability as a function of long-term trend and inter-annual variability at different locations and times^8^. For the spliced historical/future climates, estimates of trend, variability, and SNR were determined by averaging across all multi-realisation model averages.

#### Ensemble estimates of monthly temperature and precipitation

While our estimates of trend, variability, and SNR provide continuous global coverages for air temperature and precipitation from the Last Glacial Maximum to the end of the 21^st^ Century at centennial time scales, we recognise that some researchers may want to work with datasets that cover different time periods, e.g. seasonal or decadal trends. Therefore, we also provide ensemble mean estimates of monthly temperature (°C) and precipitation (mm/day) for the historical and future climates at the same spatial resolution of our continuous trend, variability, and SNR estimates. These ensembles allow end users to create their own estimates of trend, variability, and SNR at time scales suitable for their purposes (e.g. seasonal or decadal). These ensemble means are provided only for the spliced historical/future climate period (1850–2100 C.E.). We opted not to provide ensemble means for the pre-industrial control runs as these simulations are not reconstructions of temporally explicit pre-industrial climate (unlike, e.g., TraCE-21ka), but are used to simulate internal model variability, which can be used as a proxy for natural (unforced) climate variability. When combined with the data in *PaleoView*, the historical/future ensemble means provide a spatiotemporally harmonised monthly temperature and precipitation climate dataset from 21,000 B.P. to 2100 C.E. Monthly ensembles were generated using CDO^40^, by averaging across all realisations within each model, and then across models, for each of the RCP scenarios.

## Data Records

Access to *StableClim* is through figshare^54^. Dataframes for the results of the global and regional regressions under pre-industrial, past, and historical/RCP conditions are stored as data.tables^47^ in named lists in a compressed RDS format^55^. These are also provided as CSV files inside a tar.gz archive within *StableClim*. The gridded datasets have been created as NetCDF files. A geopackage containing the aggregated IPCC regions and the Wallace zoogeographic regions and realms can also be found in the ‘gpkg’ folder within *StableClim*. A tutorial showing how to extract and subset the data is also provided.

The naming convention for the results of the global and regional regressions is:

StableClim_ < *scenario* > _ < *var* > .RDS

where *scenario* is the name of the scenario (piControl, past, spliced historical), and *var* represent either global and regional regression thresholds for the pre-industrial control simulation, or the slopes for global/regional temperature regressions for the past and historical/RCP data.

The naming convention for the ensemble mean monthly data is:

StableClim_MonthlyEnsemble_ < *scenario* > _ < *var* > .nc

and for the regression files:

StableClim_Regression_ < *scenario* > _ < *var* > .nc

where *scenario* is the name of the scenario (past, spliced historical RCP 2.6–RCP 8.5), and *var* is pr (precipitation) or ts (air temperature).

The monthly ensemble temperature and precipitation have the following dimensions – 72 × latitude, 144 × longitude, 3012 × months. The units for the monthly ensembles are pr = mm/day, ts = °C. Each of the regression files contains three record variables: (1) = Trend, (2) = Variability, (3) = Signal:Noise ratio. These record variables have the following dimensions – 72 × latitude, 144 × longitude, and year [20,902 for the past, 251 for the historical/RCP]. Units for the regressions are pr = mm/year, ts = °C/year.

Multi-model *median* estimates of trend, variability, and SNR can be easily generated using the provided code. Likewise, regressions on bias corrected datasets for the past and historical/RCP simulations, can be easily generated by applying anomalies/bias corrections to the data as appropriate, before using the provided regression code.

## Technical Validation

The TraCE-21ka simulation has previously been well validated across multiple spatial and temporal scales with regards to its ability to simulate known rapid climate change events^28,34–36^, and to accurately model contemporary climates^28^. As such, we have done no additional technical validation on the raw temperature or precipitation data extracted from the TraCE-21ka simulation. Validations have, however, been done on estimates of SNR (see below for details).

The CMIP5 pre-industrial and RCP simulations are built using the same model structure as for the historical simulations but with altered forcing and boundary conditions^25^. An assessment of agreement between historical multi-model ensemble-averaged projections of temperature and precipitation, and observed temperature and precipitation provides confidence that trends, variability, and SNR measures provided in *StableClim* are an accurate representation of recent and future climates^28^.

The thresholds of extreme change we provide to subset continuous estimates of global-mean temperature trend, variability, and SNR to periods of rapid climate change have been validated recently. Brown, *et al*.^8^ identified past centuries of rapid change in global-mean temperature, over the period 21,000 B.P. to 100 B.P. as those having absolute global-mean temperature trends greater than the 90^th^ percentile of the pre-industrial control CDF. To check that their definition of rapid climate change was appropriate, they ran two tests: 1) Brown, *et al*.^8^ calculated the CDF for trends from the TraCE-21ka model and compared these to the CDF based on periods of rapid climate change from the pre-industrial control simulations; and 2) they determined the amount of time a calendar millennium was considered to be experiencing rapid rates of climate change by calculating the % of time that a millennium was characterised by trends ≥90^th^ percentile of the pre-industrial control run trends. This confirmed that known large-scale climatic events during the last deglaciation (e.g. Bølling–Allerød) were being correctly identified as periods of rapid climate change in their analysis (see Supplementary Fig. 6 in Brown, *et al*.^8^). The tacit assumption made here is that changes in grid-cell temperatures (and variability) scale approximately linearly with changes in global-mean temperature.

### Signal to noise ratio

To validate our method of calculating signal-to-noise ratio (SNR), we calculated estimates of SNR for Antarctica and Greenland using latitudinally weighted temperatures and compared these to estimates based on the Vostok^56^ and NGRIP^57,58^ ice-cores. The temporal resolution and timing of temperature estimates was matched between the TraCE-21 simulation and the ice-core data by sub-setting the (annual) TraCE-21 data to the same time steps as the Vostok (~150 years) and NGRIP data (~20 years). This allowed us to calculate estimates of SNR at centennial timescales between observed (ice-core) and simulated (TraCE-21) datasets that were directly comparable. Boxplots of SNR values for four different windows during which high and low magnitude climate fluctuations occurred at the poles (21-15k B.P.; 15-11k B.P.; 11-3k B.P.; >3k B.P.) were constructed for visual interpretation, before the SNR values were statistically compared using PERMDISP^59^ and PERMANOVA^60^ on a Euclidean distance matrix in PRIMER^61^ with the PERMANOVA+ addon^62^. The four different windows were chosen as there are known major rapid climate change events that occur within at least the first three windows: the oldest Dryas and the H1 Heinrich events occur in the period 21-15k^63–68^, the Bølling–Allerød, Antarctic Cold Reversal, Younger Dryas, and the 11.7 event occur in the period 15-11k^63,64,67,69^, and the 8.2k event occurs within the 11-3k window^70^. Both procedures had data source (TraCE-21 or ice-core) nested within window and used 999 permutations to generate P-values.

**Fig. 4:**
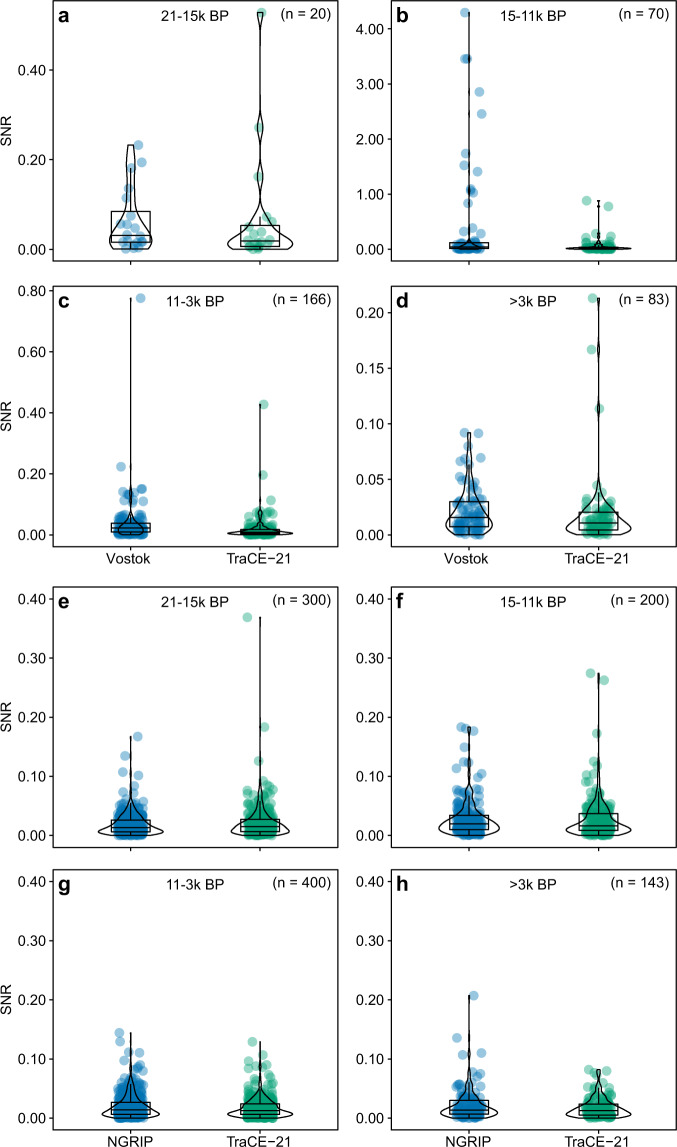
Validation of our modelled Signal-to-Noise ratio (SNR) against SNR calculated for the Vostok (**a**–**d**) and NGRIP (**e**–**h**) ice-cores^56–58^. Differences between the shape of the distributions and the SNR values were significant in **b**, with significant differences in mean SNR for **b** and **c**, but non-significant in all other windows based on PERMDISP and PERMANOVA results.

The PERMDISP results suggest there were significant differences in the dispersion of SNR values between sources (i.e. between the ice-core and simulated data) within windows for the Vostok core. However, after accounting for multiple comparisons^71^, only one of the results was considered significant (15-11k comparison, adj. P = 0.01). Likewise, unadjusted P-values were significant for comparisons between the NGRIP core and our simulated estimate of SNR, but after adjusting for multiple comparisons none of the results were considered significant (all P ≥ 0.45). These results suggest there were only significant differences in the dispersion of observed (Vostok) and simulated (TraCE-21) SNR during the period 15-11k B.P. The PERMANOVA results suggested significant differences between sources within window for the Vostok core (pseudo-F_4,670_ = 11.82, P = 0.001; Fig. 4), with pairwise comparisons confirming differences in the 15-11k (t = 3.16, adj. P = 0.008) and the 11-3k window (t = 2.81, adj. P = 0.013). There were no differences in the NGRIP ice core comparison (pseudo-F_3,4_ = 1.64, P = 0.188; Fig. 4). These results suggest significant differences in mean SNR values between the observed and simulated datasets only for the Antarctic region in the 15-11k and 11-3k windows. In other words, for the NGRIP-TraCE21 comparisons there were no statistically significant SNR differences, while for the comparisons with Vostok data, and in particular the 15-11k window, the results were more equivocal.

### Multi-model temperature and precipitation ensembles

Our multi-model ensemble climate data was validated at global and regional scales for land surfaces only, at a spatial resolution of 2.5°. To validate our ensemble mean historical temperature and precipitation datasets, we extracted gridded high resolution (0.5° × 0.5°, monthly time step) data between 1901 and 2018 from the Climatic Research Unit (CRU) time-series database^72^. The data were re-gridded to the same 2.5 × 2.5° grid of our ensemble monthly estimates and converted to annual average temperature and average total monthly precipitation. Annual average climatologies for temperature and precipitation were then calculated for both the CMIP5 ensemble-mean historical dataset and the re-gridded CRU dataset, for a 50-yr period centered on 1980, globally and for a range of different regions.

**Fig. 5:**
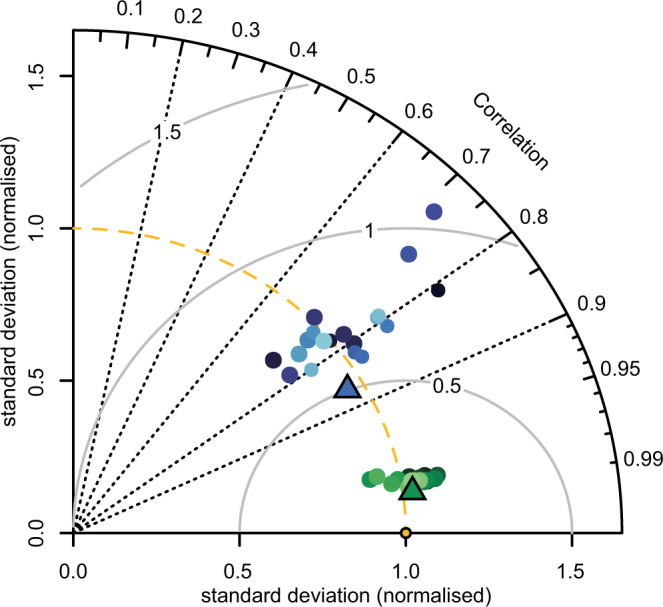
Taylor diagram showing the relationship between ensemble estimates of temperature (green points), precipitation (blue points), and the CRU TS v. 4.03 dataset (orange point) for a 50-yr period centered on 1980 calculated at global extent. Each circle represents a different model, with ensemble means shown by the triangles. The reference (CRU) climatology is shown by the orange circle, with SD values normalised to 1.

**Fig. 6:**
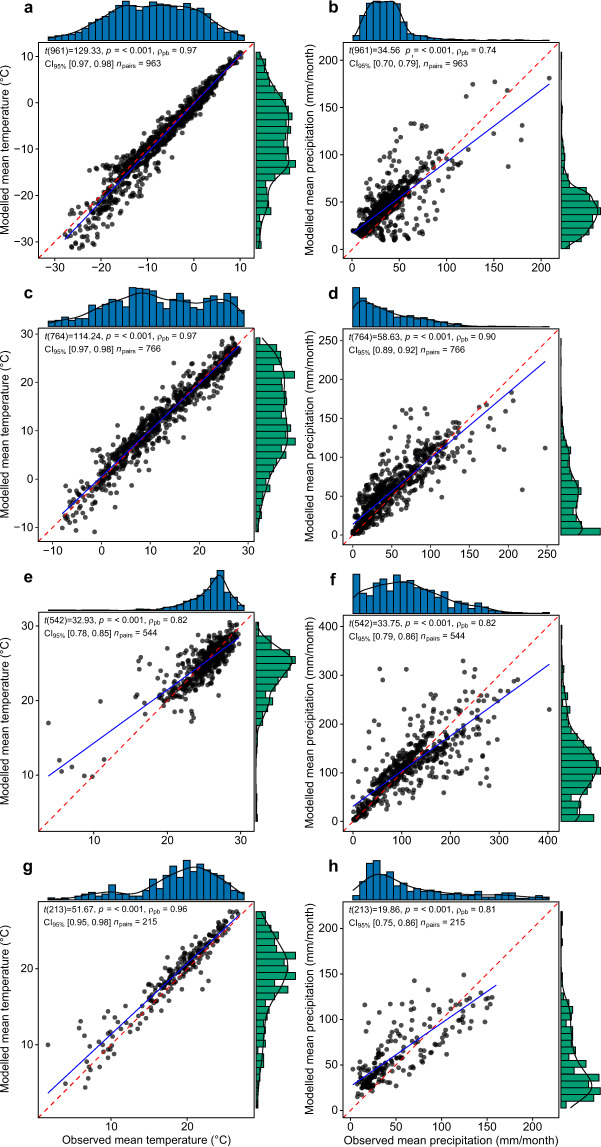
Comparison of simulated and observed historical temperatures and precipitation. Simulated data are ensemble mean estimates and observed data are from the CRU TS v. 4.03 dataset. Comparisons are shown for different latitudes for a 50-yr period centered on 1980. High-north (50°:90°; **a**,**b**), Mid-north (20°:50°; **c**,**d**), High-tropics (−20°:20°; **e**,**g**), and Mid-south (−20°:−50°; **g**,**h**). All percentage bend correlations are significant at P < 0.001.

To quantify the skill of our ensemble model to recreate observed temperature and precipitation conditions we used a combination of visual and statistical approaches. Figure 5 shows the relatively high pattern correlations and low standard deviations between our ensemble estimates and the re-gridded CRU data at a global scale^73^. The spread in inter-model correlations and standard deviations was, as expected, much higher for simulated precipitation than for temperature^74^. We also calculated a range of statistical metrics to quantify the relationship between our ensembled data and the CRU data, namely: Percentage bend correlation^75^, M-statistic^76^, latitudinally weighted Root-Mean-Square-Error, ratio-of-standard-deviations, modified index of agreement^77^, and percentage bias. These metrics were calculated globally and for four latitudinal bands: High-North (50°N–90°N), Mid-North (20°N–50°N), Mid-South (50°S–20°S) and the High-Tropics (20°S–20°N)^8,28^. Five IPCC AR5 regions^24^ and 4 biogeographic realms^25^ were also included in the validation (Table 1). All correlations were significant at P < 0.001 with correlation coefficient ranging between 0.67 (Neotropical realm) and 0.99 (Table 1, Fig. 6). The M-statistic ranged between 40.5 and 91.3, with no clear relationship between scale and the resultant score indicating the ensemble estimate of climate has varying capacities to simulate observed conditions independent of scale. Percentage bias in precipitation varied between −3.4 and 31.5% with the lowest values occurring in the tropics and the Mediterranean (Table 1). The ensemble mean precipitation was shown to over-estimate precipitation across all latitudinal bands. On average, over a range of spatial scales, precipitation was overestimated by ~11%.

**Table 1:** Metrics used to assess the ability of our ensemble estimate of historical temperatures and precipitations to replicate observed conditions.

Region	Temperature					Precipitation				
	ρ_pb_	M	RMSE_w_	rSD	md	ρ_pb_	M	%-bias	rSD	md
Global	0.99	91.3	1.98	1.03	0.95	0.89	64.5	10.9	0.93	0.76
High-North	0.97	83.3	1.99	1.08	0.89	0.75	51.5	21.0	0.98	0.61
Mid-North	0.97	84.3	2.17	0.99	0.89	0.91	63.8	15.5	0.99	0.78
Mid-South	0.96	78.2	1.70	0.97	0.85	0.81	52.8	20.9	0.90	0.65
High-Tropics	0.81	61.1	1.83	0.86	0.72	0.83	57.4	0.10	0.92	0.73
High Latitudes^*^	0.96	78.9	2.28	1.12	0.87	0.72	66.1	13.6	0.94	0.61
Mediterranean and Sahara^*^	0.96	81.2	1.53	0.87	0.85	0.97	77.1	−2.1	0.97	0.86
North America (East)^*^	0.99	89.0	0.99	0.94	0.92	0.89	62.5	6.4	0.79	0.72
Southern Africa and West Indian Ocean^*^	0.79	60.4	1.72	0.89	0.75	0.90	48.1	31.5	0.76	0.55
Australia and New Zealand^*^	0.99	78.9	1.42	0.94	0.83	0.93	66.1	13.2	0.79	0.71
Neotropical^#^	0.95	79.1	2.10	0.91	0.88	0.67	40.5	−2.1	0.77	0.59
Oriental^#^	0.86	76.3	2.34	1.05	0.81	0.84	61.9	−3.4	0.95	0.75
Palearctic^#^	0.98	86.5	2.13	1.05	0.91	0.88	57.5	22.8	0.89	0.67

ρ_pb_ = percentage bend correlation^75^, where higher values indicate more agreement between observed and simulated conditions; M = m statistic^76^ (×100), where higher values indicate more agreement between observed and simulated conditions; RMSE_w_ = Root-Mean-Square-Error weighted by latitude, lower values indicate better agreement between simulated and observed conditions; rSD = ratio of standard deviations, values closer to 1 indicate better agreement between simulated and observed conditions; md = modified index of agreement^77^, values closer to 1 indicate better agreement between simulated and observed conditions; %-bias = percentage bias, the tendency of the simulated values to be larger or smaller than observed. *IPCC AR5 regions from van Oldenborgh, *et al*.^24^. ^#^Biogeographic realms following Holt, *et al*.^25^.

## Usage Notes

To further account for the large inter-model differences in spatial resolution, forcings, physics, and sensitivities within each of the AOGCMS^43^, we recommend using pattern scaling approaches^78^ where local (cell-based) “raw” trends in temperature and precipitation are standardised by the trend in global-mean temperature for the matching window. This technique has been applied previously^8,44,51^. Due to the method of calculating Signal-to-Noise ratio we recommend inspecting the individual trend and variability components when interpreting analyses on SNR (Fig. 3). See Brown, *et al*.^8^ for an analysis which classifies trend and variability into a range of classes representing different qualitative levels of climate stability. See Supplementary File 1 for an example analysis which involves subsetting the data to periods of regionally rapid climate change, pattern scaling the trends and producing maps of trend, variability, and SNR.

## Supplementary information

Supplementary File 1

## Data Availability

Code used to generate and validate *StableClim* is available at https://github.com/GlobalEcologyLab/StableClim, with bash scripts to download the CMIP5 data from ESGF available at https://github.com/GlobalEcologyLab/ESGF_ClimateDownloads.

## Online-only Table

**Online-only Table 1:** The 20 models used for the analysis of paleoclimate from the last glacial maximum through to pre-industrialisation (21,000 B.P. – 100 B.P.), ‘natural’ climate conditions simulated with the CMIP5 pre-industrial control runs, historical climate simulations (1850–2005), and future simulated climate (2006–2100) under four Representative Concentration Pathways (2.6, 4.5, 6.0, and 8.5).

			Atmospheric Resolution (°)			
Model	Ensemble^*^	Institution/s	Lat	Long	Number of years^†^	RCP scenarios
ACCESS 1.3	r1i1p1	Commonwealth Scientific and Industrial Research Organisation / Bureau of Meteorology	1.9	1.2	500	4.5, 8.5
BCC-CSM1.1	r1i1p1	Beijing Climate Center, China Meteorological Administration	2.8	2.8	500	2.6, 4.5, 6.0, 8.5
CanESM2	r1i1p1, r2i1p1, r3i1p1, r4i1p1, r5i1p1	Canadian Centre for Climate Modelling and Analysis	2.8	2.8	996	2.6, 4.5, 8.5
CCSM4	r1i1p1, r2i1p1, r3i1p1, r4i1p1, r5i1p1, r6i1p1	National Center for Atmospheric Research	0.9	1.3	1,051	2.6, 4.5, 6.0, 8.5
CESM1(CAM5)	r1i1p1, r2i1p1, r3i1p1	National Center for Atmospheric Research	0.9	1.3	320	2.6, 4.5, 6.0, 8.5
CSIRO-Mk3.6.0	r1i1p1, r2i1p1, r3i1p1, r4i1p1, r5i1p1, r6i1p1, r7i1p1, r8i1p1, r9i1p1, r10i1p1	Commonwealth Scientific and Industrial Research Organisation / Queensland Climate Change Centre of Excellence	1.9	1.9	500	2.6, 4.5, 6.0, 8.5
GFDL-CM3	r1i1p1	National Oceanic and Atmospheric Administration Office of Oceanic and Atmospheric Research - Geophysical Fluid Dynamics Laboratory	2	2.5	500	2.6, 4.5, 6.0, 8.5
GISS-E2-H	r1i1p1, r2i1p1, r3i1p1, r4i1p1, r5i1p1, r6i1p1	NASA / GISS (Goddard Institute for Space Studies)	2.5	2	312	2.6, 4.5, 6.0, 8.5
GISS-E2-R	r1i1p1, r2i1p1, r3i1p1, r4i1p1, r5i1p1, r6i1p1	NASA / GISS (Goddard Institute for Space Studies)	2.5	2		2.6, 4.5, 6.0, 8.5
HadGEM2-CC	r1i1p1, r2i1p1, r3i1p1	Met Office Hadley Centre	1.3	1.9	240	4.5, 8.5
HadGEM2-ES	r1i1p1, r2i1p1, r3i1p1, r4i1p1	Met Office Hadley Centre	1.3	1.9	575	2.6, 4.5, 6.0, 8.5
INM-CM4	r1i1p1	Institute for Numerical Mathematics	1.5	2	500	4.5, 8.5
IPSL-CM5A-LR	r1i1p1, r2i1p1, r3i1p1, r4i1p1	Institut Pierre-Simon Laplace	1.9	3.8	1,000	2.6, 4.5, 8.5
IPSL-CM5A-MR	r1i1p1	Institut Pierre-Simon Laplace	1.3	2.5	300	2.6, 4.5, 8.5
MIROC5	r1i1p1	Atmosphere and Ocean Research Institute (The University of Tokyo), National Institute for Environmental Studies / Japan Agency for Marine-Earth Science and Technology	1.4	1.4	670	2.6, 4.5, 6.0, 8.5
MIROC-ESM	r1i1p1, r2i1p1, r3i1p1, r4i1p1, r5i1p1	Japan Agency for Marine-Earth Science and Technology, Atmosphere and Ocean Research Institute (The University of Tokyo) / National Institute for Environmental Studies	2.8	2.8	630	2.6, 4.5, 6.0, 8.5
MPI-ESM-LR	r1i1p1, r2i1p1, r3i1p1	Max Planck Institute for Meteorology	1.9	1.9	1,000	2.6, 4.5, 8.5
MRI-CGCM3	r1i1p1	Meteorological Research Institute	1.1	1.1	500	2.6, 4.5, 6.0, 8.5
NorESM1-M	r1i1p1	Norwegian Climate Centre	1.9	2.5	501	2.6, 4.5, 6.0, 8.5
TraCE-21ka^#^	N/A	National Center for Atmospheric Research	2.5	2.5	21,000	

^*^All pre-industrial control simulations only utilised ensemble r1i1p1. See methods for details. ^†^Number of years of pre-industrial control simulation.

All historical/RCP simulations were spliced to the matching realisation^42^. ^#^TraCE-21ka data was pre-processed for PaleoView^28^.
