# Supplementary material for: StableClim, continuous projections of climate stability from 21000 BP to 2100 CE at multiple spatial scales
Source: Sci Data. 2020 Oct 12;7:335. doi: 10.1038/s41597-020-00663-3 (PMC7550347; doi:10.1038/s41597-020-00663-3)
Supplement: Supplementary file 1 [file 41597_2020_663_MOESM1_ESM.pdf]

StableClim - Supplementary File 1

Stuart C. Brown, Tom M. L. Wigley, Bette L. Otto-Bliesner, Damien A. Fordham

August 2020

Contents

Load in *StableClim* **2**

Subsetting *StableClim* **4**

Applying Thresholds **5**

Subsetting raster data **7**

Masking **8**

Rates of climate change in Eurasia **8**

Plotting **9**

In this appendix we'll quickly explore how to subset the data contained in the *StableClim* database to periods of extreme regional warming for the Eurasian Wallace Zoogeographic region.

Load in *StableClim*

Here we read in some of the necessary R libraries for opening and exploring the *StableClim* database. We then import the lists of dataframes that contain the regional thresholds, and finally read in the geopackage containing the regions we are interested in working with.

```r
# Load libs
library(data.table)
library(sf)
library(raster)
library(ggplot2)

# Thresholds
piThresh <- readRDS("./StableClim_piControl_thresholds.RDS")

# Past (TraCE-21ka) regressions
pastReg <- readRDS("./StableClim_past_RegionalTemperatureRegressions.RDS")

# Past regression rasters
pastRastTrend <- brick("./ncdf/regressions/StableClim_Regression_past_ts.nc",
  varname = "ts_trend"
)
pastRastVar <- brick("./ncdf/regressions/StableClim_Regression_past_ts.nc",
  varname = "ts_variability"
)
pastRastSNR <- brick("./ncdf/regressions/StableClim_Regression_past_ts.nc",
  varname = "ts_snr"
)

# Historical and future regressions
futReg <- readRDS("./StableClim_HistoricalRCP_RegionalTemperatureRegressions.RDS")

# Historical/Future rasters under RCP 2.6
futRastTrend <- brick("./ncdf/regressions/StableClim_Regression_rcp26_ts.nc",
  varname = "ts_trend"
)
futRastVar <- brick("./ncdf/regressions/StableClim_Regression_rcp26_ts.nc",
  varname = "ts_variability"
)
futRastSNR <- brick("./ncdf/regressions/StableClim_Regression_rcp26_ts.nc",
  varname = "ts_snr"
)

# Geopackage containing the regions
regionalShp <- read_sf("./gpkg/StableClim_VectorData.gpkg", layer = "WallaceRegions")

## subset the regionalShp to Eurasia, and union to single multipolygon
eurasia <- st_union(regionalShp[regionalShp$Name == "Eurasian", ])
```

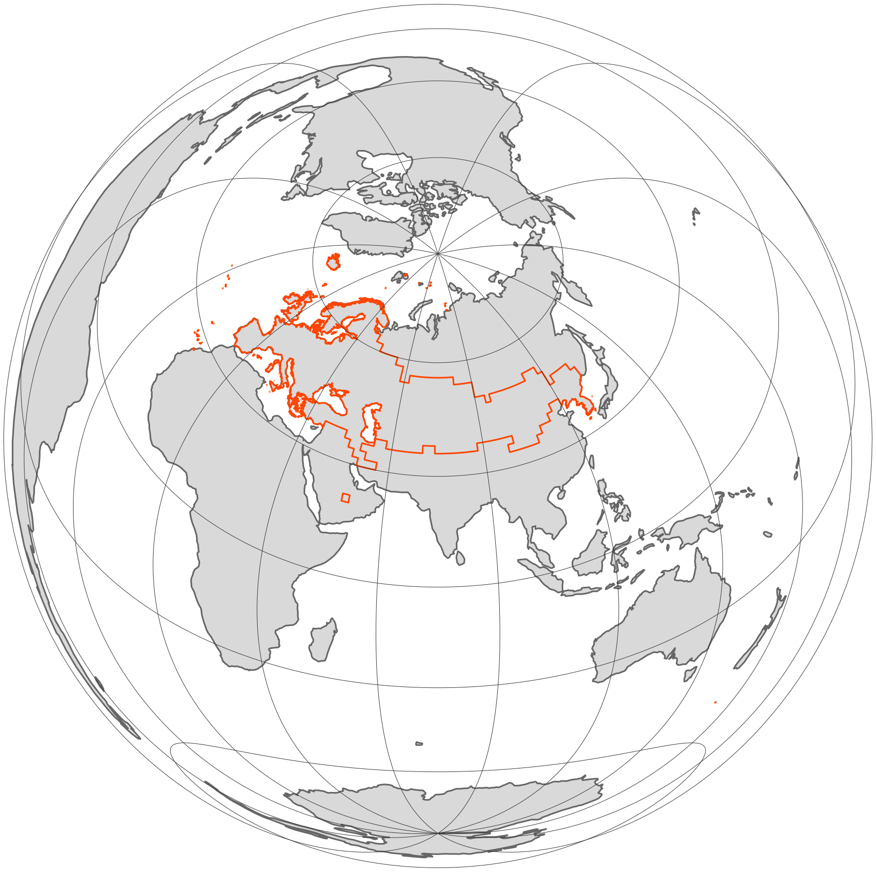

Figure 1: Map of the Eurasian Wallace Zoogeographic region

Subsetting *StableClim*

Looking at names of the `piThresh` list, we can see there are four dataframes stored in the `piThresh` object.

```
names(piThresh)
```

```
## [1] "All Periods [absolute]" "All Periods [signed]"   "Cool periods"
## [4] "Warm periods"
```

We're only interested in working with warming periods, so we can subset the `piThresh` list to `warming periods`.

```
warming_periods <- piThresh[["Warm periods"]]
```

A quick look at the `warming_periods` data.table shows how the thresholds for climate change are organised in the table. We can see that the first column desribes the region type (e.g. Wallace region), the second column describes the region of interest (e.g. Eurasian), while the remaining columns describe the thresholds of climate change in 1, 2.5, and 5% increments.

Table 1: A quick look at the warming periods. Note, here only some of the columns are shown and the thresholds have been multiplied by 100.

RegionType	Region	1%	2.5%	5%	10%	90%	95%	97.5%	99%
Global	Land	0.00177	0.00459	0.00842	0.01688	0.20861	0.23997	0.25869	0.27505
Global	Land/Sea	0.00160	0.00384	0.00739	0.01349	0.16806	0.19009	0.20471	0.21360
Global	Ocean	0.00188	0.00349	0.00690	0.01368	0.16048	0.18139	0.19912	0.21075
Holt realms	Afrotropical	0.00144	0.00286	0.00619	0.01240	0.14417	0.17168	0.18924	0.20667
Holt realms	Australian	0.00300	0.00609	0.01154	0.02164	0.27985	0.32842	0.36875	0.39080
Holt realms	Madagascan	0.00254	0.00472	0.00831	0.01682	0.19936	0.22400	0.24558	0.26328
Holt realms	Nearctic	0.00391	0.00791	0.01462	0.02670	0.31263	0.35901	0.39700	0.42101
Holt realms	Neotropical	0.00250	0.00468	0.00837	0.01656	0.16921	0.19327	0.21586	0.23931
Holt realms	Oceanian	0.00128	0.00287	0.00550	0.01055	0.10412	0.11786	0.13133	0.14148
Holt realms	Oriental	0.00161	0.00360	0.00710	0.01531	0.15766	0.17979	0.20028	0.21624

We can then extract the threshold we're interested in, for example 90%, using the following code

```
thresh <- warming_periods[RegionType == "Wallace" & Region == "Eurasian", ][["90%"]]
## threshold in °C/Year
thresh
```

```
## [1] 0.003804441
```

This threshold shows us that we would expect extreme warming (90th percentile) 'natural climate variability' to be characterised by temperature changes of 0.38 °C/century in the Eurasian zoogeographic region.

Applying Thresholds

Now that we know our threshold of rapid climate change we can subset the past and the historical/future data to these periods only. This is a two step process:

1. Subset the regional regressions, and extract a list of windows which are $\geq$ the threshold
2. Subset the rasters for the respective time periods to windows $\geq$ the threshold.

A quick look at the names of the `pastReg` and `futReg` lists shows that they are a little more complex than the `piThresh` list.

```
names(pastReg)[1:10]
```

```
##  [1] "Global.Land/Sea"          "Global.Land"
##  [3] "Global.Ocean"             "Wallace.South American"
##  [5] "Wallace.Australian"       "Wallace.Novozelandic"
##  [7] "Wallace.African"          "Wallace.Madagascan"
##  [9] "Wallace.Papua-Melanesian" "Wallace.Amazonian"
```

```
names(futReg)[1:10]
```

```
##  [1] "rcp26.Global.Land/Sea"          "rcp26.Global.Land"
##  [3] "rcp26.Global.Ocean"             "rcp26.Wallace.South American"
##  [5] "rcp26.Wallace.Australian"       "rcp26.Wallace.Novozelandic"
##  [7] "rcp26.Wallace.African"          "rcp26.Wallace.Madagascan"
##  [9] "rcp26.Wallace.Papua-Melanesian" "rcp26.Wallace.Amazonian"
```

We need to subset the `pastReg` list based on `RegionType.Region`, and the `futReg` list based on `scenario.RegionType.Region`

```
pastReg <- pastReg[["Wallace.Eurasian"]]
```

Table 2: A quick look at regional regression results for the Eurasian region simulated by TraCE-21.

Scenario	Start	End	RegionType	Region	GMT	Slope	Var	Method
TraCE-21ka	-21000	-20901	Wallace	Eurasian	-4.35	-0.00431	0.42441	GLS
TraCE-21ka	-20999	-20900	Wallace	Eurasian	-4.36	-0.00366	0.42273	GLS
TraCE-21ka	-20998	-20899	Wallace	Eurasian	-4.35	-0.00350	0.42570	GLS
TraCE-21ka	-20997	-20898	Wallace	Eurasian	-4.35	-0.00324	0.43018	GLS
TraCE-21ka	-20996	-20897	Wallace	Eurasian	-4.35	-0.00315	0.43075	GLS
TraCE-21ka	-20995	-20896	Wallace	Eurasian	-4.35	-0.00393	0.43427	GLS
TraCE-21ka	-20994	-20895	Wallace	Eurasian	-4.35	-0.00378	0.43460	GLS
TraCE-21ka	-20993	-20894	Wallace	Eurasian	-4.35	-0.00355	0.43524	GLS
TraCE-21ka	-20992	-20893	Wallace	Eurasian	-4.35	-0.00348	0.43569	GLS
TraCE-21ka	-20991	-20892	Wallace	Eurasian	-4.34	-0.00320	0.44132	GLS

```
futReg <- futReg[["rcp26.Wallace.Eurasian"]]
```

Table 3: A quick look at regional regression results for the Eurasian region simulated by our RCP 2.6 future ensemble.

Scenario	Start	End	RegionType	Region	GMT	Slope	Var	Method
rcp26	1850	1949	Wallace	Eurasian	6.52	0.00245	0.18898	GLS
rcp26	1851	1950	Wallace	Eurasian	6.53	0.00228	0.18905	GLS
rcp26	1852	1951	Wallace	Eurasian	6.53	0.00266	0.18837	GLS
rcp26	1853	1952	Wallace	Eurasian	6.53	0.00247	0.18825	GLS
rcp26	1854	1953	Wallace	Eurasian	6.53	0.00272	0.18715	GLS
rcp26	1855	1954	Wallace	Eurasian	6.53	0.00261	0.18723	GLS
rcp26	1856	1955	Wallace	Eurasian	6.54	0.00314	0.18704	GLS
rcp26	1857	1956	Wallace	Eurasian	6.54	0.00383	0.18883	GLS
rcp26	1858	1957	Wallace	Eurasian	6.55	0.00293	0.18385	GLS
rcp26	1859	1958	Wallace	Eurasian	6.55	0.00253	0.18324	GLS

Here we can see we have a 9 column data.table returned. The columns are:

1. Scenario, which describes the climate scenario
2. Start, the start year of the window of analysis
3. End, the end year of the window of analysis
4. RegionType, the type of region
5. Region, the specific region relevant to `RegionType`
6. GMT, the weighted regional mean temperature (dependent on `Region`) for the window
7. Slope, the slope of a regression model fit to annual weighted global mean temperatures over the window
8. Var, the *S.D.* of the residuals of the regression model
9. Method, the type of regression (either GLS or OLS. See manuscript for details.)

We can now subset the `pastReg` and `futReg` data.tables based on our threshold of extreme climate change.

```
pastReg_RCC <- pastReg[pastReg$Slope >= thresh, ]
futReg_RCC <- futReg[futReg$Slope >= thresh, ]
```

The `pastReg_RCC` data.table now contains only 2392 rows, from a total of 20901 rows previously. Likewise, the `futReg_RCC` data.table contains 120 rows, from a total of 152 suggesting for the Eurasian zone, more often that not (~ 79 % of the time) rates of climate change since wide-scale industrialisation (~1850 C.E.) have been greater than expected under extremely high levels of natural climate variability.

Using the `Start` column from the `pastReg_RCC` data.table we can create a vector of layer names which can be matched to the names of the `pastRast` raster brick we imported earlier.

Subsetting raster data

We can the use this subset raster to calculate median rates of extreme climate change given the threshold.

```
# Check the names of the raster
names(pastRastTrend)[1:10]
```

```
##  [1] "X.21000" "X.20999" "X.20998" "X.20997" "X.20996" "X.20995" "X.20994"
##  [8] "X.20993" "X.20992" "X.20991"
```

```
# make Start names the same format as the layer names
layerIDX <- gsub("-", "X\\.", pastReg_RCC$Start)
layerIDX <- which(names(pastRastTrend) %in% layerIDX)

# Subset the raster data, and calculate median values through time
rcc_past_trend <- calc(readAll(pastRastTrend[[layerIDX]]), median)
rcc_past_var <- calc(readAll(pastRastVar[[layerIDX]]), median)
rcc_past_snr <- calc(readAll(pastRastSNR[[layerIDX]]), median)
```

Masking

Now that we have the rasters subset to the correct time periods and we have calculated the median rates of climate change, we can crop and mask the raster data to the Eurasian region and then plot it.

In the usage notes of the manuscript we also advocate using a pattern scaled trend. We can calculate this here too and then recalculate SNR using the pattern scaled trend.

For masking and cropping we first transform the `eurasia` polygons into a raster. As of writing this appendix, `sf` objects don't play nicely with rasters, so we can either `rasterize` the polygons or turn them to `sp` polygon objects. Here we first convert to `SpatialPolygons`, then `rasterize`, and then remove cells that have less than 10% coverage in Eurasia.

```
# convert the Eurasia region to `sp` and then to raster
eurasia_raster <- rasterize(as(eurasia, "Spatial"),
  y = raster(res = res(rcc_past_trend)),
  getCover = TRUE
)

# set areas that are less than 10% inside Eurasia to NA
eurasia_raster[eurasia_raster < 0.10] <- NA

# Mask the trend, variability, and SNR
## multiply trend * 100 to get °C/century if required
eurasia_trend <- mask(rcc_past_trend, eurasia_raster)
eurasia_var <- mask(rcc_past_var, eurasia_raster)
eurasia_snr <- mask(rcc_past_snr, eurasia_raster)

# create pattern scaled trends and recalc SNR
# use the mean of the the regional trends for the scaling
## N.B. if trend calculated above is * 100,
## mean(pastReg)$Slope also needs to be * 100 for pattern scaling.
eurasia_trendPS <- eurasia_trend / mean(pastReg$Slope)
eurasia_snrPS <- abs(eurasia_trendPS) / eurasia_var
```

Rates of climate change in Eurasia

From the `eurasia_trend` raster layer for Eurasia we can see that the minimum and maximum rates of median climate change during periods of rapid warming were 0.14 °C/century and 1.42 °C/century.

```
eurasia_trend * 100
```

```
## class      : RasterLayer
## dimensions : 72, 144, 10368  (nrow, ncol, ncell)
## resolution : 2.5, 2.5  (x, y)
## extent     : -180, 180, -90, 90  (xmin, xmax, ymin, ymax)
## crs        : +proj=longlat +datum=WGS84 +ellps=WGS84 +towgs84=0,0,0
## source     : memory
## names      : layer
## values     : 0.1429243, 1.418756  (min, max)
```

Plotting

We can now plot maps of the Eurasian region showing trend, variability, and SNR for the past.

NB - Here we plot the non-pattern scaled trend in °C/Century.

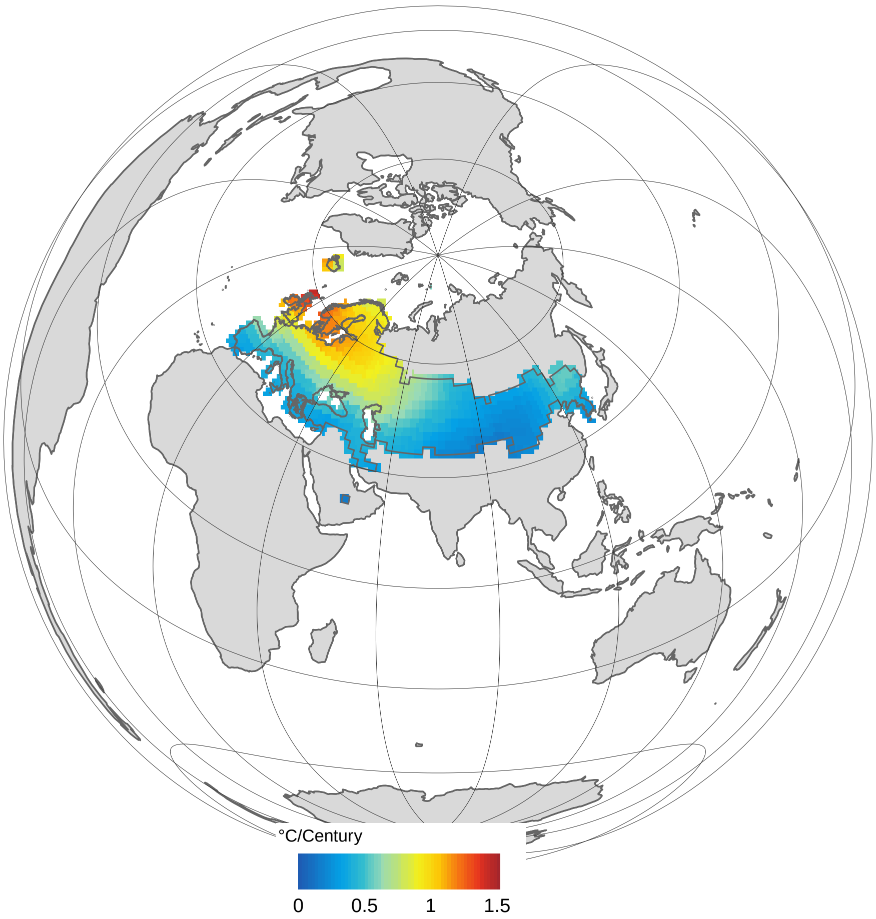

Figure 2: Plot of centennial trend in temperature for the past.

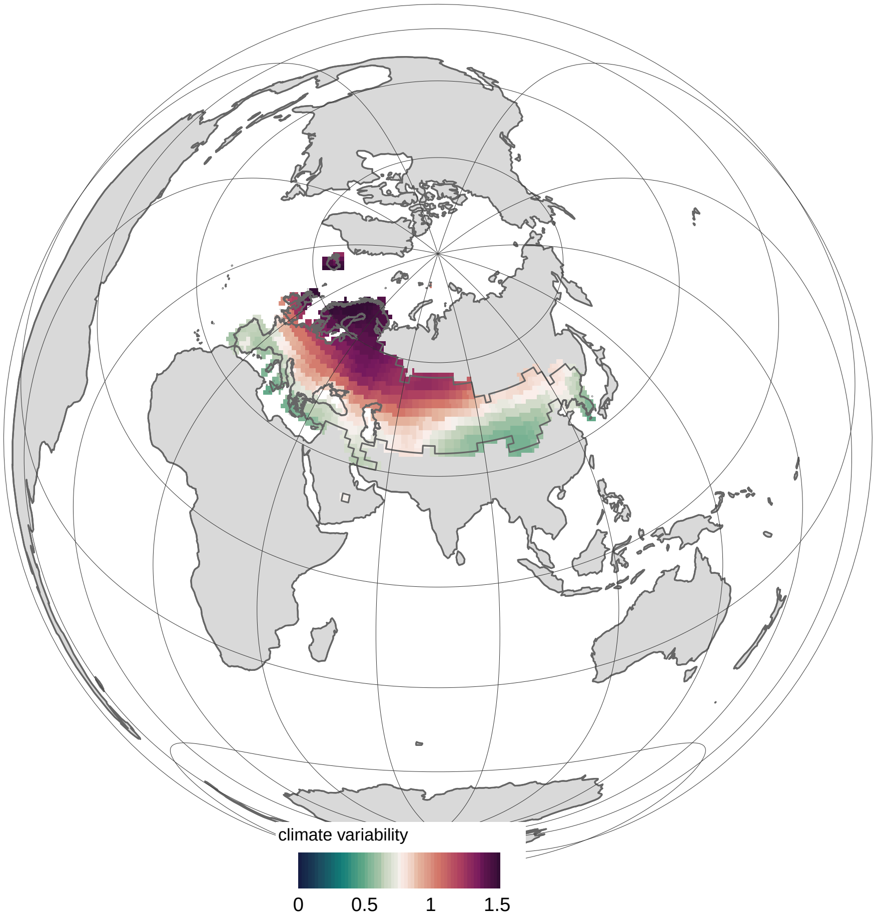

Figure 3: Plot of temperature variability for the past.

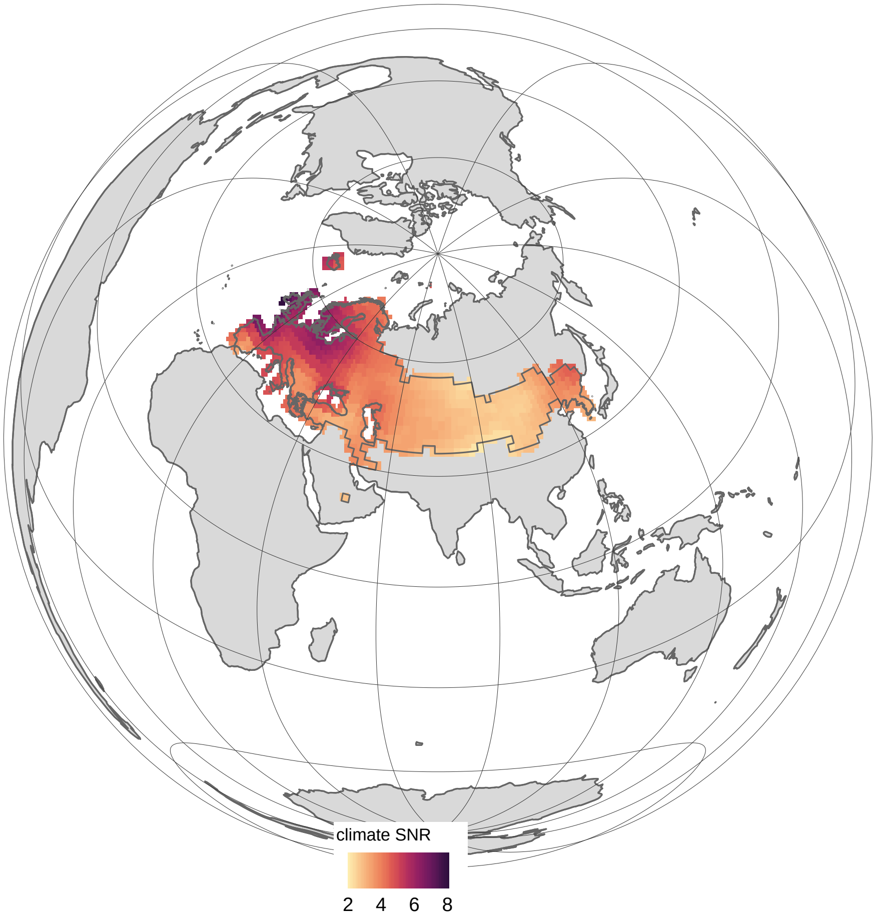

Figure 4: Plot of temperature SNR for the past.

We will leave it up to you to create the necessary rasters for the future period, using the steps above.

```r
sessionInfo()
```

```
## R version 3.5.1 (2018-07-02)
## Platform: x86_64-w64-mingw32/x64 (64-bit)
## Running under: Windows 10 x64 (build 18362)
##
## Matrix products: default
##
## locale:
## [1] LC_COLLATE=English_Australia.1252  LC_CTYPE=English_Australia.1252
## [3] LC_MONETARY=English_Australia.1252 LC_NUMERIC=C
## [5] LC_TIME=English_Australia.1252
##
## attached base packages:
## [1] stats     graphics  grDevices utils     datasets  methods   base
##
## other attached packages:
## [1] tmap_3.0          ggplot2_3.1.0     raster_3.1-6      sp_1.4-1
## [5] sf_0.9-2          data.table_1.12.8
##
## loaded via a namespace (and not attached):
##  [1] Rcpp_1.0.4.6       lattice_0.20-35    png_0.1-7          class_7.3-14
##  [5] assertthat_0.2.1   digest_0.6.25      mime_0.9           R6_2.3.0
##  [9] plyr_1.8.4         backports_1.1.3    evaluate_0.14      e1071_1.7-1
## [13] highr_0.8          pillar_1.3.1       rlang_0.4.5        lazyeval_0.2.1
## [17] rstudioapi_0.8     rmarkdown_2.1      styler_1.0.2       rgdal_1.4-8
## [21] stringr_1.4.0      htmlwidgets_1.3    munsell_0.5.0      shiny_1.2.0
## [25] compiler_3.5.1     httpuv_1.4.5       xfun_0.13          base64enc_0.1-3
## [29] pkgconfig_2.0.2    tmaptools_3.0      rgeos_0.4-2        htmltools_0.4.0
## [33] tidyselect_1.0.0   tibble_2.1.1       codetools_0.2-15   XML_3.98-1.16
## [37] viridisLite_0.3.0  crayon_1.3.4       dplyr_0.8.3        withr_2.1.2
## [41] later_0.7.5        grid_3.5.1         lwgeom_0.2-3       xtable_1.8-3
## [45] gtable_0.2.0       DBI_1.0.0          magrittr_1.5       units_0.6-1
## [49] scales_1.0.0       ncdf4_1.16         KernSmooth_2.23-15 stringi_1.4.6
## [53] promises_1.0.1     leafsync_0.1.0     leaflet_2.0.2      RColorBrewer_1.1-2
## [57] rematch2_2.0.1     tools_3.5.1        dichromat_2.0-0    leafem_0.1.1
## [61] glue_1.4.0         purrr_0.3.3        crosstalk_1.0.0    abind_1.4-5
## [65] parallel_3.5.1     yaml_2.2.1         colorspace_1.3-2   stars_0.4-1
## [69] classInt_0.4-3     knitr_1.28
```
